# Machine learning based on metabolomics reveals potential targets and biomarkers for primary Sjogren’s syndrome

**DOI:** 10.3389/fmolb.2022.913325

**Published:** 2022-09-05

**Authors:** Kai Wang, Ju Li, Deqian Meng, Zhongyuan Zhang, Shanshan Liu

**Affiliations:** Department of Rheumatology, The Affiliated Huaian No.1 People’s Hospital of Nanjing Medical University, Huaian, China

**Keywords:** primary Sjogren’s syndrome, systemic lupus erythematosus, metabolomics, machine learning, 2-hydroxypalmitic acid, L-carnitine, cyclic AMP

## Abstract

**Background:** Using machine learning based on metabolomics, this study aimed to construct an effective primary Sjogren’s syndrome (pSS) diagnostics model and reveal the potential targets and biomarkers of pSS.

**Methods:** From a total of 39 patients with pSS and 38 healthy controls (HCs), serum specimens were collected. The samples were analyzed by ultra-high-performance liquid chromatography coupled with high-resolution mass spectrometry. Three machine learning algorithms, including the least absolute shrinkage and selection operator (LASSO), random forest (RF), and extreme gradient boosting (XGBoost), were used to build the pSS diagnosis models. Afterward, four machine learning methods were used to reduce the dimensionality of the metabolomics data. Finally, metabolites with significant differences were screened and pathway analysis was conducted.

**Results:** The area under the curve (AUC), sensitivity, and specificity of LASSO, RF and XGBoost test set all reached 1.00. Orthogonal partial least squares discriminant analysis was used to classify the metabolomics data. By combining the results of the univariate false discovery rate and the importance of the variable in projection, we identified 21 significantly different metabolites. Using these 21 metabolites for diagnostic modeling, the AUC, sensitivity, and specificity of LASSO, RF, and XGBoost all reached 1.00. Metabolic pathway analysis revealed that these 21 metabolites are highly correlated with amino acid and lipid metabolisms. On the basis of 21 metabolites, we screened the important variables in the models. Further, five common variables were obtained by intersecting the important variables of three models. Based on these five common variables, the AUC, sensitivity, and specificity of LASSO, RF, and XGBoost all reached 1.00.2-Hydroxypalmitic acid, L-carnitine and cyclic AMP were found to be potential targets and specific biomarkers for pSS.

**Conclusion:** The combination of machine learning and metabolomics can accurately distinguish between patients with pSS and HCs. 2-Hydroxypalmitic acid, L-carnitine and cyclic AMP were potential targets and biomarkers for pSS.

## Introduction

Primary Sjogren’s syndrome (pSS), a chronic inflammatory autoimmune disease of unknown etiology, is characterized by salivary and lacrimal gland hypofunction. SS can be a disease that is primary or secondary to other autoimmune diseases, such as rheumatoid arthritis, systemic lupus erythematosus (SLE), and systemic sclerosis. There is a strong relationship between pSS and human leukocyte antigens (HLA), *IRF5-TNPO3, STAT4, PTPN22, IL12A, FAM167A-BLK, DDX6-CXCR5,* and *TNIP1* (Lessard et al., 2013). Although these genes are related to pSS, any mutation of them will not result in the development of pSS. Environmental factors may also be implicated in the development of pSS. As a result of the consistent increase chromium in the soil, there is an increase in the prevalence of pSS (Lee et al., 2019). Metabolomics is a useful tool for the identification of changes in metabolic pathways, including abnormal downstream changes in small molecule metabolites due to changes in upstream protein-encoding genes and changes in metabolites due to environmental factors. Metabolomics is used to improve the understanding of the origin and pathogenesis of disease.

In recent years, metabolomics has provided new insights into the pathogenesis of pSS; it has great potential in identifying new biomarkers of pSS and shows the potential of metabolomics in diagnosing pSS. A metabolomics study based on urine and serum of patients with pSS revealed that the changes in the primary metabolic pathway in patients with pSS were related to the metabolism of phospholipids, fatty acids, and amino acids (tryptophan proline and phenylalanine) (Fernández-Ochoa et al., 2020). The results of saliva metabolomics revealed that the distribution diversity of metabolites in the saliva of patients with pSS was lower than that of healthy controls (HCs), and that the metabolite distribution of patients with pSS was affected by salivary adenitis (Kageyama et al., 2015). Notwithstanding, is there any evidence supporting the pSS-specificity of the differential metabolites? Unfortunately, the presence of these metabolites was not confirmed in other rheumatic diseases by the above-mentioned studies.

Therefore, in this study, we analyzed the serum from patients with pSS and HCs using ultra-performance liquid chromatography coupled with high-resolution mass spectrometry (UPLC-HRMS) and processed the metabolomics data using a machine learning approach in order to discover potential diagnostic biomarkers in the serum. Afterward, we verified the potential diagnostic biomarkers in SLE, thereby revealing the potential correlation between serum metabolites and the development of pSS.

## Materials and methods

### Study designing, and participants

The study design is shown in Supplementary Figure S1. A total of 39 patients newly diagnosed with pSS were recruited according to the revised American-European Consensus Group classification criteria for pSS (Shiboski et al., 2017). Patients with a history of radiotherapy administered to the neck, head, and face; patients with hepatitis C virus infection, AIDS, lymphoma, sarcoidosis, and Graves’ disease; and patients using anti-acetylcholine drugs were excluded from the study. All patients were Chinese and were hospitalized at the Affiliated Huaian No.1 People’s Hospital of Nanjing Medical University. A total of 38 age-, sex-, and race-matched HCs constituted the control group. The clinical information of the patients and HCs were recorded (Table 1). An additional 11 serum samples, including five HCs and six patients with pSS, were collected as the indepengdent validation cohort (Supplementary Table S1). The inclusion-exclusion criteria for the indepengdent validation cohort are the same as mentioned above. Previous pSS metabolomics studies have not further validated the expression of the identified biomarkers in other rheumatic diseases (Urbanski et al., 2021; Xu et al., 2021). Therefore, this study also recruited a total of 44 patients with SLE and age-, sex-, and race-matched HCs in order to validate whether the potential biomarkers identified for pSS are specific (Supplementary Table S2). All subjects avoided strenuous exercise and excitant drink 1 day before the sample collection and 5 ml of venous blood was collected in coagulant tubes in the morning under the fasting state. The study was approved by the Ethics Committee of the Affiliated Huaian No.1 People’s Hospital of Nanjing Medical University. In accordance with the Declaration of Helsinki, all participants were informed about the purpose of the study.

**TABLE 1:** Demographics and clinical characteristics of pSS and HCs.

Characteristics	HCs (*n* = 38)	pSS (*n* = 39)
Age (mean, range)	36.50 ± 9.82	37.62 ± 12.72
Gender (F/M)	30/8	33/6
Cholesterol (mmol/L)	4.01 ± 1.09	5.61 ± 1.46^a^
ESR (mm/h)	17.06 ± 3.47	30.61 ± 8.14^a^
CRP (mg/L)	4.76 ± 1.77	12.00 ± 4.24^a^
Anti-SSA Antibody (positive/negative)	0/38	29/10^a^
Anti-SSB Antibody (positive/negative)	0/38	13/26^a^
ANA (positive/negative)	2/36	26/13^a^

a *p* < 0.05 for Wilcoxon test for pSS, patients and HCs.

Abbreviations: pSS, primary Sjogren’s syndrome; ESR, erythrocyte sedimentation rate; CRP, C-reactive protein; HCs, Healthy controls.

### Sample preparation

For protein precipitation, samples (10 μL) were taken from each group and cold methanol (30 μL, 4°C) was added, vortexed for 30 s, and centrifuged at 16,000 g in a freeze centrifuge for 15 min at 4°C. Using a centrifugal concentration dryer (Labconco, United States), the supernatant was collected and evaporated at room temperature. The residue was reconstituted in pure methanol (20 μL). By replacing the serum with ultra-pure water and applying the same standard operating procedure for serum samples, extraction blanks were prepared.

### Conditions of UPLC-HRMS

Chromatographic analysis was conducted with a Hypersil GOLD C_18_ (100 mm × 2.1 mm, 1.9 μm, Thermo Scientific, Germany) column on a UPLC Ultimate 3,000 system (Dionex, Germering, Germany) coupled to a Q-Exactive mass spectrometer (QEMS, Thermo Fisher Scientific, Bremen, Germany) in both positive and negative modes simultaneously. The autosampler and column temperatures were set to 4°C and 40°C, respectively, and the injection volume was 5 µL. Binary gradient elution (channel A: acetonitrile, 0.1% v/v formic acid, channel B: ultra-pure water, 0.1% v/v formic acid) was done at a flow rate of 400 μL/min over a run time of 15 min with the gradient elution program as follows: 0–3 min, 99% B; 3–10 min, 99% B; 10–13 min, 1% B; 13–13.1 min, 1% B; 13.1–15 min, 99% B. To avoid complications related to the injection order, all samples were analyzed in a randomized fashion. Using a QEMS equipped with a heated electrospray ionization source, the MS data were collected. For both positive and negative modes, the operating parameters were as follows: a spray voltage of 3.5 kV and 2.5 kV for the positive and negative modes, respectively, a capillary temperature of 250°C, a sheath gas flow of 50 arbitrary units, an auxiliary gas flow of 13 arbitrary units, a sweep gas of 0 arbitrary units, and an S-lens RF level of 60. The resolution was set at 70,000 in the full-scan analysis (70–1,050 m/z). Following the manufacturer’s instructions, the MS system was calibrated. Chemical identification was performed based on the retention time and the accurate mass of commercial standards.

### Metabolite identification

Metabolite ion peaks were extracted using the R package XCMS with the following parameters: mass accuracy: 25 ppm, peak width (5, 25), snthresh: 12, prefilter: (5, 5,000). For overlapping peaks, the minimum difference in m/z was 7.5 mda. The closest approach was used for grouping before and after retention time (RT) correction, with 9 s as the rtCheck parameter and an allowable RT difference of 2.5%. Finally, the original data files of the missing peak regions were reintegrated using the fillPeaks method, thus filling in the missing data points. Peak area, RT and peak width were extracted from the XCMS data and UPLC-HRMS features for each sample. Metabolic structure identification uses mass accuracy and secondary spectrogram matching to search the self-built compound’s library. The self-built compounds’ library was established from 500 metabolite standards (≥98.0%, Sigma-Aldrich, St. Louis, MO, United States). The final library contains 415 endogenous metabolites, which are mainly enriched in glucose metabolism, lipid metabolism, amino acid metabolism, nucleotide metabolism and phenolic metabolism.

### Data analysis

The computational analysis was done using Python (version 3.6.2, Python Software Foundation, Delaware, United States) and R (version 4.0.5, Foundation for Statistical Computing, Vienna, Austria). The Mann - Whitney U tests were used to compare the characteristics of the two groups of subjects. *P* values less than 0.05 were considered to statistically significant.

### Machine learning approach

To establish diagnostic models of pSS, three machine learning algorithms were applied: least absolute shrinkage and selection operator (LASSO), random forest (RF), and extreme gradient boosting (XGBoost). These machine algorithms (LASSO, RF, and XGBoost) were applied using the R package glmnet (Friedman et al., 2010), ranger (Wright and Ziegler, 2017) and xgboost (Chen and Guestrin, 2016), respectively. Metabolomics data was divided into two parts: the training set (70%) and the test set (30%). We performed a repeated K-fold cross-validation (repeats = 5, K = 5) on the models in order to obtain an unbiased estimate for their performance. Using the R package pROC, the receiver operating characteristic (ROC) curve was prepared (Robin et al., 2011). Using the R package ComplexHeatmap, heatmap analysis was performed (Gu et al., 2016).

Metabolomics data are characterized by high dimensionality and small samples sizes. Moreover, many redundant metabolites are capable of causing curse of dimensionality, interfering with diagnosis, and reduce the accuracy of classifiers. Therefore, to reduce the dimensionality of the metabolomics data, different methods were adopted. Among these methods, the principal component analysis (PCA), an unsupervised method of data dimensionality reduction, can visually describe the differences in metabolic patterns and clustering results between different groups and identify the original variables that contribut to the intergroup classification as biomarkers using load maps. PCA was performed using the Python package PCA. Another method adopted is the use of a variational autoencoder (VAE), which is an unsupervised learning technique that uses artificial neural networks to learn low-dimension features from high-dimension features, thereby enabling the mapping of data points in the original high-dimensional space to a low-dimensional space. Keras (version 2.3.1) with TensorFlow (version 1.15.0) backend was used to construct the VAE model. The partial least squares-discriminant analysis (PLS-DA) is among the most frequently used classification methods for metabolomics data analysis. It combines a regression model with dimensionality reduction and uses a certain discriminant threshold for discriminant analysis of the regression results. Furthermore, another commonly used method in metabolomics data analysis is the orthogonal projections to latent structures discriminant analysis (OPLS-DA), an extension of PLS-DA. This analysis was performed using the R package ropls (predI = 1, permI = 500, crossvalI = 7) (Thévenot et al., 2015). The variable importance in the projection (VIP) indicates the projected importance of variables in the PLS-DA and OPLS-DA models, thereby assessing the relevance of variables’ effect on the differences between the groups. Metabolite variables with VIP values >1 are considered different. To screen significantly different metabolites, we used a VIP value >1.5 combined with a false discovery rate (FDR) value <0.05.

### Biological functions of significantly different metabolites

The metabolic analysis software MetaboAnalyst 5.0 was used to analyze the metabolic pathways and biological relevance of the differential metabolites by consulting databases, such as the small molecule pathway database (SMPDB) (Jewison et al., 2014), and the Kyoto Encyclopedia of Genes and Genomes (KEGG) database (Pang et al., 2020).

## Results

### Characteristics of the study participants

The main characteristics of patients with pSS and HCs are tabulated in Table 1 (validation cohort were listed in Supplementary Table S1). There was no significant difference between pSS and HCs in terms of gender and age (*p* > 0.05). Patients with pSS had significantly higher erythrocyte sedimentation rate (ESR), C-reactive protein (CRP) and cholesterol levels and higher positive rates of anti-SSA antibodies, anti-SSB antibodies and anti-nuclear antibody (ANA) than HCs (*p* < 0.05). The main characteristics of patients with SLE and HCs are tabulated in Supplementary Table S2. There was no significant difference between SLE and HCs in gender, age and cholesterol (*p* > 0.05). Patients with SLE had significantly higher ESR and CRP levels and higher positive rates of anti-SSA antibodies, anti-SSB antibodies and ANA than HCs (*p* < 0.05).

### Metabolite identification

**FIGURE 1:**
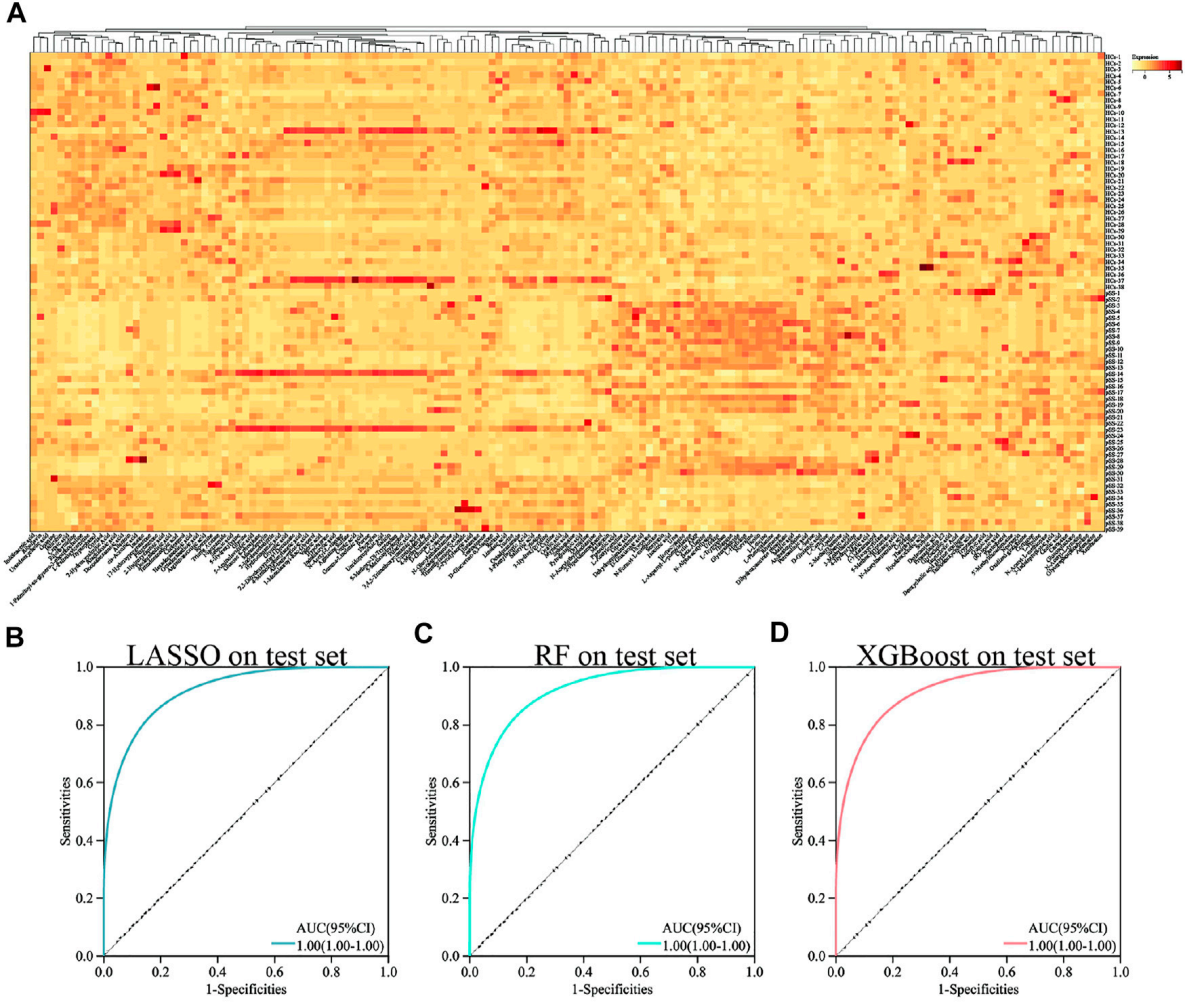
Heat map and ROC curve of different machine learning methods in all metabolites. Heat map of all metabolites **(A)**. ROC curve of LASSO in all metabolites **(B)**. ROC curve of RF in all metabolites **(C)**. ROC curve of XGBoost in all metabolites **(D)**.

After HPLC-HRMS analysis, the final number of detectable metabolites in the serum samples was 157. Figure 1A showed the overall view of all metabolites.

### Machine learning approach

**FIGURE 2:**
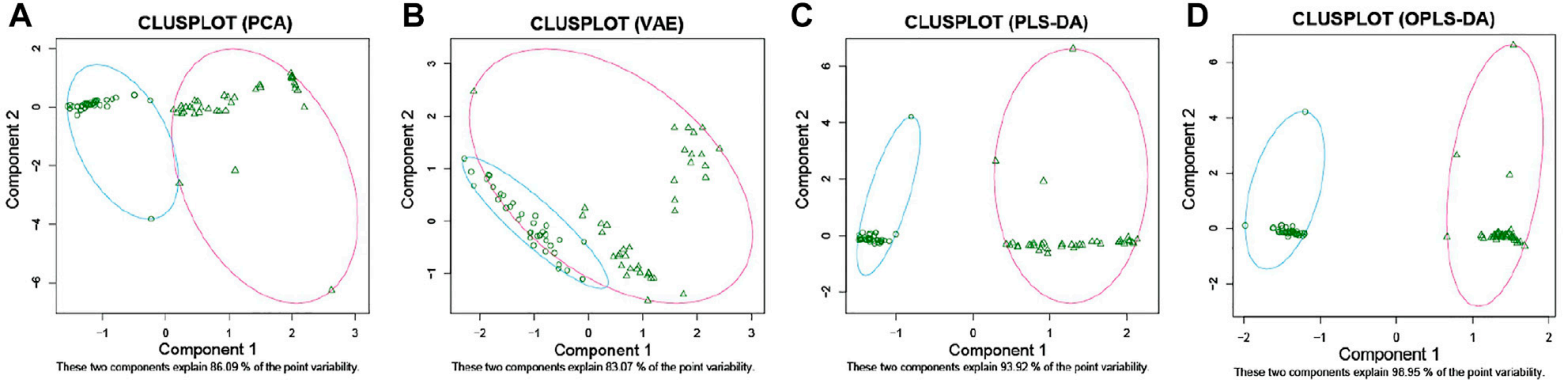
2D clustering plot based on PCA, VAE, PLS-DA, and OPLS-DA. PCA **(A)**. VAE **(B)**. PLS-DA **(C)**. OPLS-DA **(D)**.

**FIGURE 3:**
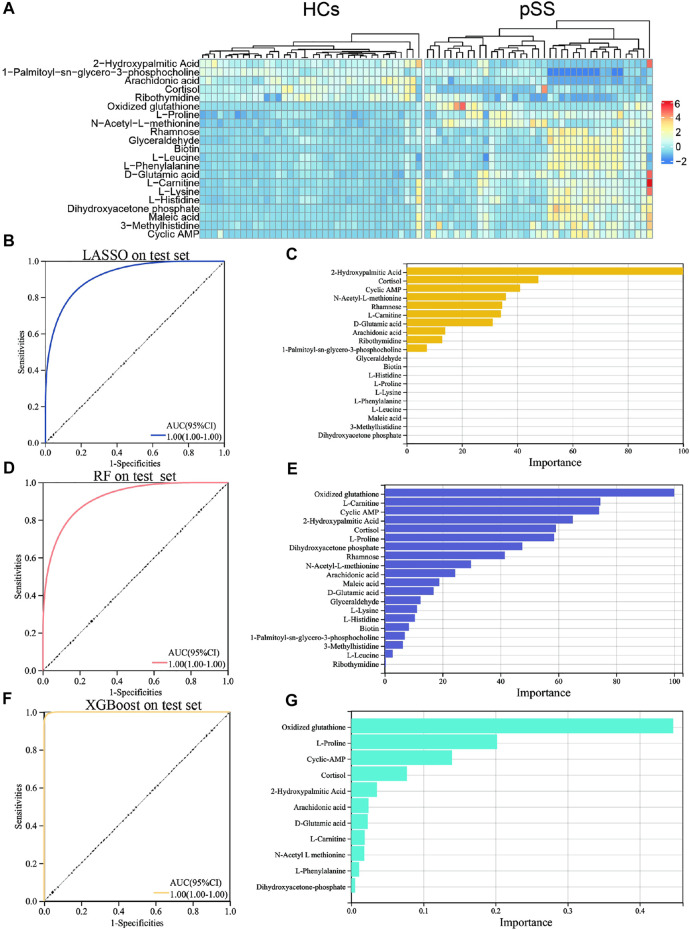
Heat map, ROC curve and important variables screening of machine learning algorithms in significantly different metabolites. Heat map of metabolites with significant differences in pSS group and HCs group **(A)**. ROC curve of LASSO model in 21 significantly different metabolites on the test set **(B)**. Important variable screening of LASSO **(C)**. ROC curve of RF model in 21 significantly different metabolites on the test set **(D)**. Important variable screening of LASSO **(E)**. ROC curve of XGBoost model in 21 significantly different metabolites on the test set **(F)**. Important variable screening of XGBoos **(G)**.

To establish a diagnostic model of pSS, we adopted three machine learning methods. The AUC, sensitivity, and specificity of LASSO and XGBoost reached 1.0. The AUC of RF was 0.982, while the sensitivity and specificity were 1.000 and 0.963, respectively (Supplementary Table S3). The AUC, sensitivity, and specificity of LASSO, RF and XGBoost test set reached 1.0 (Figures 1B–D). Different methods were applied to reduce the dimensionality of the metabolomics data. The PCA, VAE and PLS-DA methods were not satisfactory after the dimensionality reduction. As a result, the OPLS-DA method was used to classify the metabolomics data (Figure 2). In the OPLS-DA model, there was 21 metabolites with FDR and VIP values <0.05 and >1.5, respectively (Table 2). Figure 3A showed the heatmap of the 21 metabolites. Serum concentrations of these 21 metabolites were significantly different between the patients with pSS and HCs, such that the concentrations of 1-Palmitoyl-sn-glycero-3-phosphocholine, 2-Hydroxypalmitic acid, arachidonic acid, cortisol and ribothymidine in patients with pSS were lower when compared to those in HCs, while the concentrations of the other metabolites were significantly higher in patients with pSS when compared to HCs (Supplementary Figure S2, Table 2).

**TABLE 2:** The significantly different metabolites of pSS and HCs.

Metabolite	VIP	FDR	AUC	Change	HMDB	CAS
1-Palmitoyl-sn-glycero-3-phosphocholine	1.84	0.00	0.83	Down	—	17364-16-8
2-Hydroxypalmitic acid	1.63	0.00	0.91	Down	—	764-67-0
3-Methylhistidine	1.60	0.00	0.79	Up	HMDB00479	368-16-1
Arachidonic acid	2.33	0.00	0.89	Down	HMDB01043	506-32-1
Biotin	1.68	0.00	0.89	Up	HMDB00030	58-85-5
Cortisol	1.98	0.00	0.91	Down	HMDB00063	50-23-7
Cyclic AMP	2.10	0.00	0.95	Up	HMDB00058	60-92-4
D-Glutamic acid	1.85	0.00	0.87	Up	HMDB03339	6893-26-1
Dihydroxyacetone phosphate	1.79	0.00	0.91	Up	HMDB01473	—
Glyceraldehyde	1.65	0.00	0.78	Up	HMDB01051	56-82-6
L-Carnitine	1.69	0.00	0.95	Up	HMDB00062	541–15-1
L-Histidine	1.71	0.00	0.82	Up	HMDB00177	71-00-1
L-Leucine	1.66	0.00	0.8	Up	HMDB00687	61-90-5
L-Lysine	1.83	0.00	0.91	Up	HMDB00182	56-87-1
L-Phenylalanine	1.71	0.00	0.79	Up	HMDB00159	63-91-2
L-Proline	2.39	0.00	0.92	Up	HMDB00162	147-85-3
Maleic acid	1.62	0.00	0.87	Up	HMDB00176	110-16-7
N-Acetyl-L-methionine	2.15	0.00	0.92	Up	HMDB11745	65-82-7
Oxidized glutathione	1.57	0.00	0.97	Up	HMDB03337	27025-41-8
Rhamnose	1.77	0.00	0.83	Up	HMDB00849	3615-41-6
Ribothymidine	1.68	0.00	0.78	Down	HMDB00884	1463-10-1

Abbreviations: pSS, primary Sjogren’s syndrome; HCs, healthy controls; VIP, variable influence on projection; FDR, false discovery rate; HMDB, human metabolomics database; CAS, chemical abstracts service.

Supplementary Figure S3 shows the correlation between these metabolites. LASSO, RF, and XGBoost also trained the dataset of these 21 metabolites. The results of the second round of machine learning were similar to those of the first round (Figures 3B,D,F). On the basis of the second round of machine learning, we screened out the important variables in the models. There were 10 important variables in LASSO, as shown in Figure 3C. Figure 3E showed the top 20 important variables in RF. There are 11 important variables in XGBoost, as shown in Figure 3G. The third round of machine learning was based on the important variables of each model, and the results were shown in the Supplementary Table S5. We further intersect the important variables of three models to get six common variables, namely cyclic AMP (cAMP), cortisol, 2-Hydroxypalmitic acid, arachidonic acid, L-Carnitine and D-Glutamic acid (Supplementary Figure S5). The fourth round of machine learning is based on these six common variables. As shown in the Supplementary Table S6, XGBoost has the best performance. In addition, we provide the AUC plots of XGBoost (Supplementary Figure S6), and the performance summary of RF and LASSO (Supplementary Table S6).

The second round of machine learning was based on 21 metabolites with FDR and VIP values <0.05 and >1.5. If the threshold of VIP values was set to 2.0, four metabolites would be screened, namely arachidonic acid, cAMP, L-Proline and N-Acetyl-L-methionine. As shown in Supplementary Table S7, the performance of the machine learning models based on these four metabolites was lower than the results of the fourth round of machine learning. In the fourth round of machine learning, we used six metabolites, and next, we used metabolites with the top six VIP values and top six AUC values to develop the machine learning models, respectively, and the results are shown in Supplementary Tables S8,S9, and the performance of the models was still lower than the results of the fourth round of machine learning. In summary, the using of machine learning algorithms brings some advantages over the single use of OPLS-DA. Our data and code for this study can be accessed from GitHub at https://github.com/morrosun/Primary-Sjogrens-syndrome.

### Biological functions of the significantly different metabolites

**FIGURE 4:**
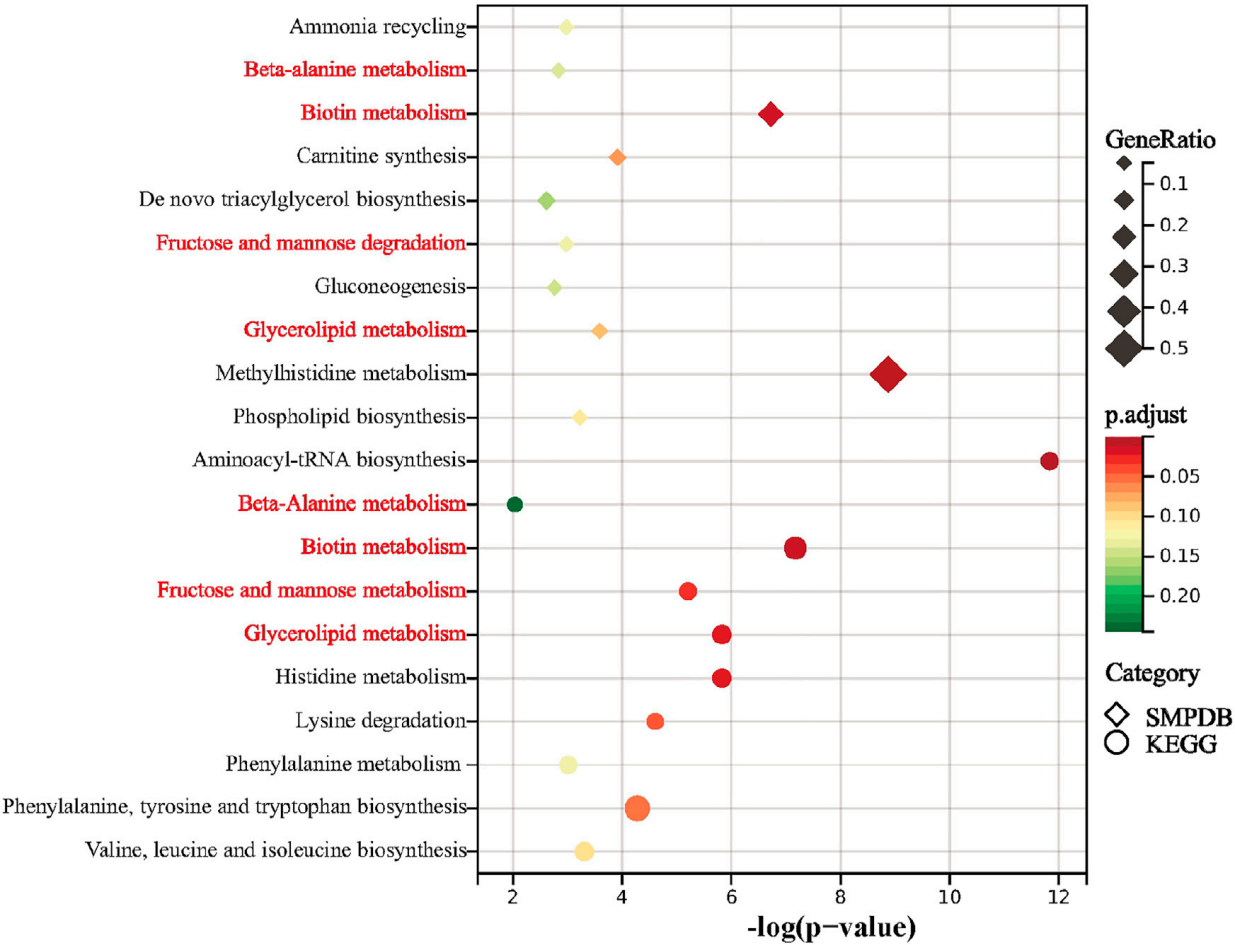
Summary plot for KEGG and SMPDB. Metabolic pathway analysis of pSS-altered metabolites using MetaboAnalyst 5.0 based on the KEGG and SMPDB. Triangles and circles colors indicate pathway enrichment significance, triangles and circles size indicates the extent of pathway impact, common pathways are marked in red.

Results of the KEGG pathway enrichment analysis revealed differential metabolites over-represented in biological processes that are mainly related to aminoacyl-tRNA biosynthesis, biotin metabolism, histidine metabolism, glycerolipid metabolism, fructose and mannose metabolism, lysine degradation, phenylalanine, tyrosine and tryptophan biosynthesis, valine, leucine and isoleucine biosynthesis, phenylalanine metabolism and beta-Alanine metabolism (Figure 4). The top 10 biological processes indicated by the SMPDB pathway enrichment analysis included methylhistidine metabolism, biotin metabolism, carnitine synthesis, glycerolipid metabolism, phospholipid biosynthesis, ammonia recycling, fructose and nannose degradation, beta-Alanine metabolism, gluconeogenesis, and *de novo* triacylglycerol biosynthesis (Figure 4).

### Definition of the potential metabolic biomarker for pSS

The AUC values of six common important variables of three machine learning models namely cAMP, cortisol, 2-Hydroxypalmitic acid, arachidonic acid, L-Carnitine and D-Glutamic acid were 0.945, 0.908, 0.904, 0.893, 0.949, and 0.672, respectively (Table 3). Compared to ANA, anti-SSA antibody, and anti-SSB antibody, cAMP, cortisol, 2-Hydroxypalmitic acid, arachidonic acid and L-Carnitine had better AUC values which suggests that these metabolites were potential biomarkers for pSS (Table 3).

**TABLE 3:** ROC curve analysis of clinical characteristics and six common variables.

	AUC	95% CI	Sensitivity (%)	Specificity (%)	*P*-Value
cAMP	0.945	0.868–0.984	92.31	89.47	<0.0001
Cortisol	0.908	0.820–0.962	87.18	92.11	<0.0001
2-Hydroxypalmitic acid	0.904	0.815–0.959	82.05	97.37	<0.0001
Arachidonic acid	0.893	0.802–0.952	89.74	71.05	<0.0001
L-Carnitine	0.949	0.874–0.986	87.18	94.74	<0.0001
D-Glutamic acid	0.672	0.561–0.771	75.00	62.50	0.005
Anti-SSA antibody	0.872	0.776–0.937	74.36	100	<0.0001
Anti-SSB antibody	0.667	0.550–0.770	33.33	100	<0.001
ANA	0.807	0.701–0.888	66.67	94.74	<0.001

Abbreviations: ROC, receiver operating characteristic; AUC, area under the curve; pSS, primary Sjogren’s syndrome; HCs, healthy controls; CI, confidence interval.

### Validation of potential biomarkers of pSS in the SLE cohort

**FIGURE 5:**
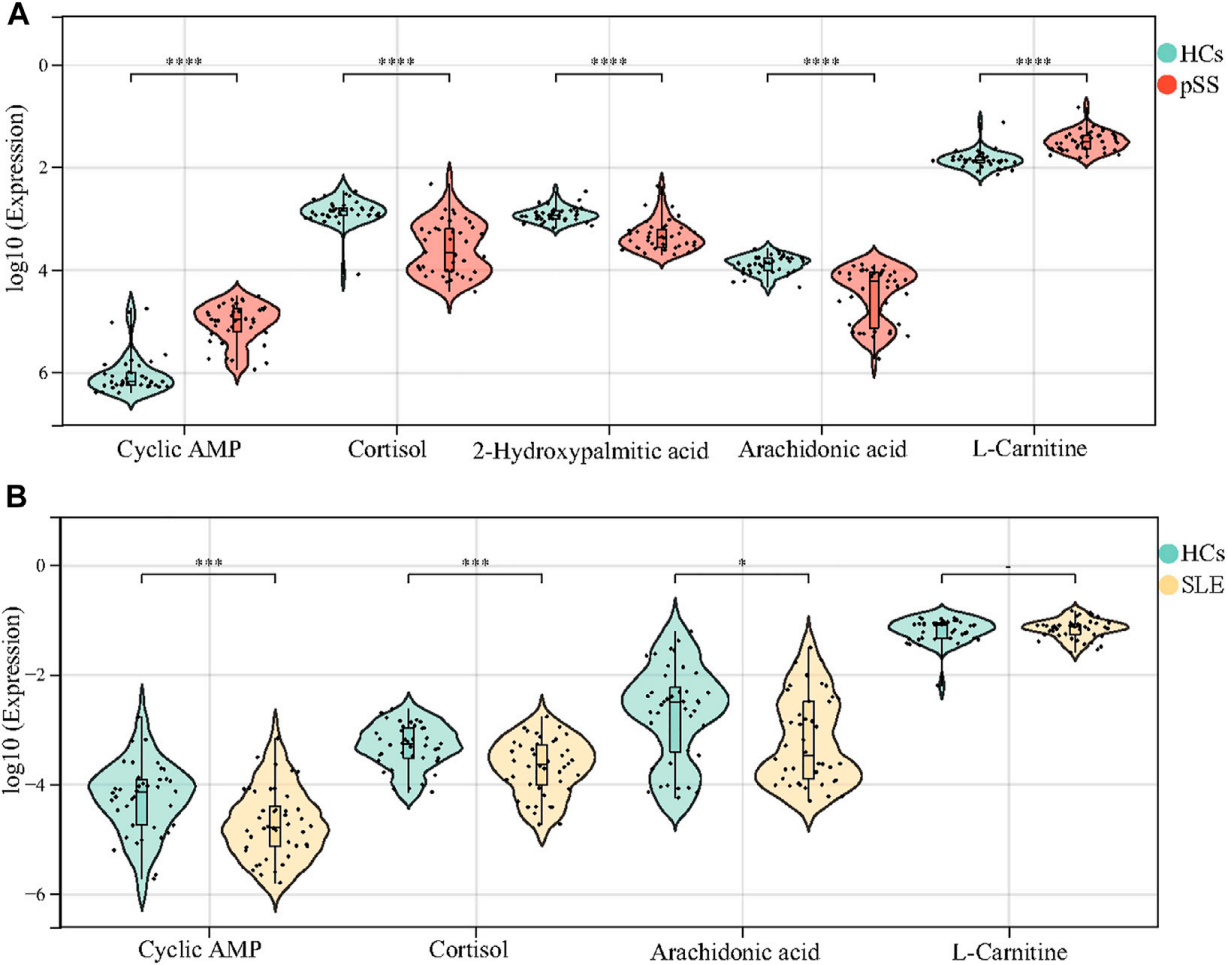
Validation of cAMP, cortisol, 2-Hydroxypalmitic acid, arachidonic acid and L-Carnitine in the SLE cohort. The levels of cAMP and L-Carnitine in patients with pSS decreased significantly compared with HCs, while the levels of cortisol, 2-Hydroxypalmitic acid and arachidonic acid inceased **(A)**. 2-Hydroxypalmitic acid was not in the metabolic profile of the SLE cohort. The levels of cAMP, cortisol and arachidonic acid in patients with SLE increased significantly compared with HCs, but there was no difference in the level of L-Carnitine **(B)**.

In this study, we revealed many significant different metabolites between patients with pSS and HCs. However, it is yet to be determined whether these differential metabolites are specific to pSS. Previous studies of pSS metabolomics have not confirmed the expression of differential metabolites in other rheumatic diseases (Urbanski et al., 2021; Xu et al., 2021). We further validated the potential markers of pSS in the SLE cohort, namely cAMP, cortisol, 2-Hydroxypalmitic acid, arachidonic acid and L-Carnitine. According to the results, a total of 129 metabolites were identified in the metabolomic analysis of SLE cohort; however, 2-Hydroxypalmitic acid was not in the metabolic profile of the SLE cohort (Supplementary Figure S7). There were no difference in the levels of L-carnitine between patients with SLE and HCs, whereas the levels of L-carnitine were significantly increased in patients with pSS (Figure 5); the level of cAMP decreased in patients with SLE and increased in patients with pSS relative to HCs (Figure 5). The levels of both cortisol and arachidonic acid were significantly decreased in patients with SLE compared to HCs, which is consistent with the results of the pSS cohort (Figure 5). The above results suggest that 2-Hydroxypalmitic acid, cAMP, and L-Carnitine were specific biomarkers of pSS.

## Discussion

Primary Sjogren’s syndrome is among the most common autoimmune diseases, affecting about 0.1–0.4% of the general population, especially in women, with a prevalence comparable to that of rheumatoid arthritis (Voulgarelis and Tzioufas, 2010). In addition to the most common salivary and lacrimal gland involvement, pSS may affect several organs, including the lungs, heart, liver, nervous system, kidneys, and joints, thus severely reducing the patients’ quality of life (Bak et al., 2017; Narvaez et al., 2020; Zhao et al., 2020). Metabolomics, as an emerging strategy, is an effective method for detecting and monitoring various diseases. The use of metabolomics data has revealed many vital relationships that are linked with affected metabolic signaling pathways that can identify and explore the centers of these networks, as well as provide opportunities for the development of novel therapeutic approaches. By integrating metabolomics and genomics data, we can create a more robust analytical tool and discover new biomarkers and compounds for targeted therapies.

Machine learning algorithms are vital in the construction of multivariate metabolite predictions. While PLS-DA and OPLS-DA have been used as classical algorithms for binary classification in metabolomics, more nonlinear machine learning methods are being applied to the study of metabolomics, such as RF, LASSO, XGBoost, support vector machines, and artificial neural networks, are being employed to study metabolomics (Yu et al., 2017; Liu et al., 2019; Mendez et al., 2019; Zhao et al., 2019; Mazzilli et al., 2020). Currently, there is no consensus regarding which algorithm is more suitable for studying metabolomics. In a previous study, the predictive performance of eight different machine learning algorithms was compared to 10 published metabolomics data-sets and it was revealed that the quality of the metabolomics data has more influence on the generalized performance than the model selection. Therefore, we used multiple machine learning algorithms to classify the pSS metabolomics data set and construct diagnostic models. We found that the predictive performance of LASSO and RF was more substantial than that of XGBoost and that the classification performance of OPLS-DA was better than that of PCA, VAE, and PLS-DA.

In this study, we revealed many significantly differential metabolites between patients with pSS and HCs; however, it is yet to be determined whether these differential metabolites are specific to pSS. Previous studies of pSS metabolomics have not confirmed the expression of differential metabolites in other rheumatic diseases. In addition to pSS, we investigated the serum metabolomics in patients with SLE and found that the levels of cAMP and L-Carnitine in patients with pSS are not consistent with those in patients with SLE, which suggests that patients with pSS have a unique metabolic profile.

Several studies have reported blood lipids changes in patients with pSS. A recent study showed that patients with pSS had higher levels of phosphatidylcholine and triglycerides but lower levels of acylcarnitine; pSS-related metabolic disorders might be related to lipid oxidation, fatty acid oxidation, and energy metabolism, and in addition, this study showed that acylcarnitine is a specific biomarker for pSS (Lu et al., 2021). Among the free fatty acids, saturated fatty acids have been demonstrated to induce inflammation via the innate immune system and participate in the pathogenesis of pSS (Shikama et al., 2017). Improving the lipid profile may serve as a new strategy for the treatment of pSS. In our study, compared to the HCs, the levels cholesterol and 2-Hydroxypalmitic acid increased in patients with pSS and the level of L-carnitine decreased. To date there have been no studies on 2-Hydroxypalmitic acid in rheumatic diseases, and our results showed that the metabolic profile of the SLE cohort does not contain 2-Hydroxypalmitic acid, suggesting that 2-Hydroxypalmitic acid is specific to pSS. The AUC of 2-Hydroxypalmitic acid was 0.904, with a sensitivity of 82.05% and a specificity of 97.37% in distinguishing patients with pSS from HCs.

L-carnitine is a kind of amino acid that promotes the conversion of fat into energy. The decrease of L-carnitine in the serum of patients with pSS indicates that the lipid metabolism pathway may have been altered. Supplementation with L-carnitine increases blood acylcarnitine levels, enhances fatty acid metabolism, and improves disorders of lipid metabolism, especially at doses above 1,500 mg/day (Wu et al., 2015; Asadi et al., 2020; Kido et al., 2020). Our results showed no difference in L-carnitine levels between patients with SLE and HCs, whereas L-carnitine levels were significantly decreased in pSS patients. L-carnitine is a specific biomarker for pSS, and the AUC of L-carnitine in distinguishing patients with pSS from HCs was 0.949, with a sensitivity of 87.18% and a specificity of 94.74%. L-carnitine supplementation may be beneficial to patients with pSS. Prospective research was needed to further confirm this result.

Further metabolomic studies also demonstrated that several lipid metabolites, such as 1-palmitoyl-sn-glycero-3-phosphocholine, 2-hydroxypalmitic acid, glyceraldehyde, and arachidonic acid, differed significantly between patients with pSS and HCs. Arachidonic acid is an unsaturated omega-6 fatty acid. A recent study showed a negative correlation between omega-6 and interleukin-21 (Castrejón-Morales et al., 2020). The lower levels of arachidonic acid may be as a result of insufficient intake (Castrejón-Morales et al., 2020). Appropriate omega-6 supplementation can improve the signs on the ocular surface, as well as the symptoms of ocular discomfort presented by patients with pSS (Aragona et al., 2005).

Cyclic AMP is an important substance involved in the regulation of substance metabolism and biological functions in cells. In our study, cyclic AMP increased in patients with SLE and decreased in patients with pSS relative to HCs, suggesting that cyclic AMP is a specific biomarker for pSS, and the AUC of cyclic AMP in distinguishing patients with pSS from HCs was 0.945, with a sensitivity of 92.31% and a specificity of 89.47%. Previous studies have shown that IgG from pSS patients could modify the biological effects mediated by activation of muscarinic choline receptor, that is, decrease cyclic AMP without affecting cyclic GMP (Bacman et al., 1996). Both mRNA and protein levels of cyclic AMP decreased significantly in SS mouse submandibular glands and increased after treatment (Wang et al., 2020). Activation of the cyclic AMP/protein kinase A pathway upregulates the expression of the water channel aquaporin 5 in the apical plasma membranes of submandibular gland acinar cells, which plays a critical role in regulating salivary flow rates (Saito et al., 2015). These results are consistent with our findings, indicating that cyclic AMP may be an important target in the treatment of pSS.

Our study adopted a machine learning approach to investigate UPLC-HRMS based on serum metabolomics data and developed high sensitivity and specificity models to diagnose pSS. At the same time, our study screened the significantly different metabolites in the serum of patients with pSS. Moreover, changes in the biological pathways of these metabolites may be helpful to understand the potential mechanism underlying the pathogenesis of pSS. Compared to HCs, the metabolites of patients with pSS in the metabolic network changed significantly and correlated with each other (Supplementary Figure S3). The majority of the metabolic pathways in the metabolic network were related to the metabolism of fatty acids and amino acids. Our results need to be further validated and the exact mechanisms need to be further investigated.

## Data Availability

The raw data supporting the conclusions of this article will be made available by the authors, without undue reservation.
