# Supplementary material for: Machine learning based on metabolomics reveals potential targets and biomarkers for primary Sjogren’s syndrome
Source: Front Mol Biosci. 2022 Sep 5;9:913325. doi: 10.3389/fmolb.2022.913325 (PMC9483105; doi:10.3389/fmolb.2022.913325)
Supplement: Supplementary file 1 [file DataSheet1.docx]

**
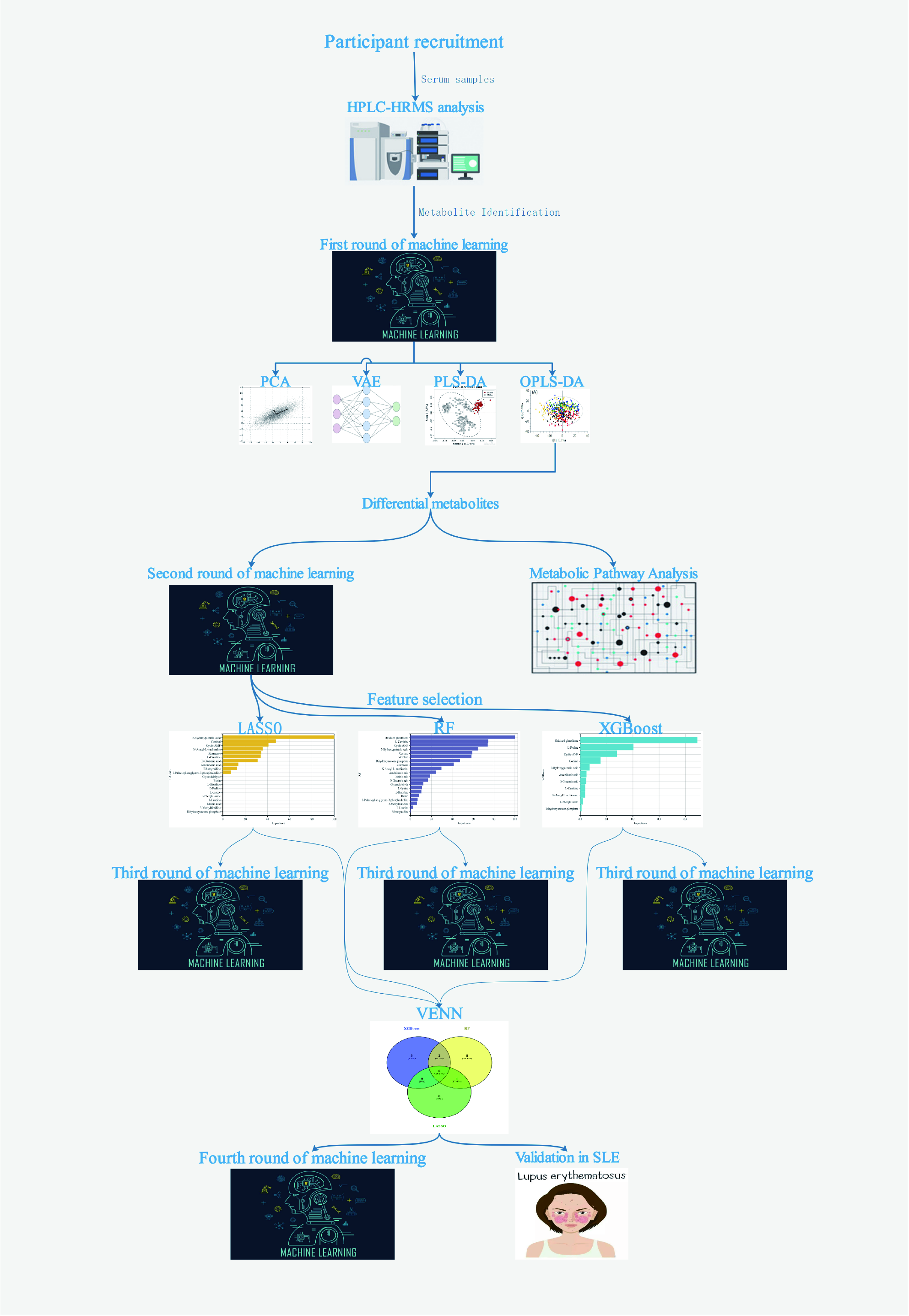
**

**Supplementary Figure S1**. The flow chart of the study.


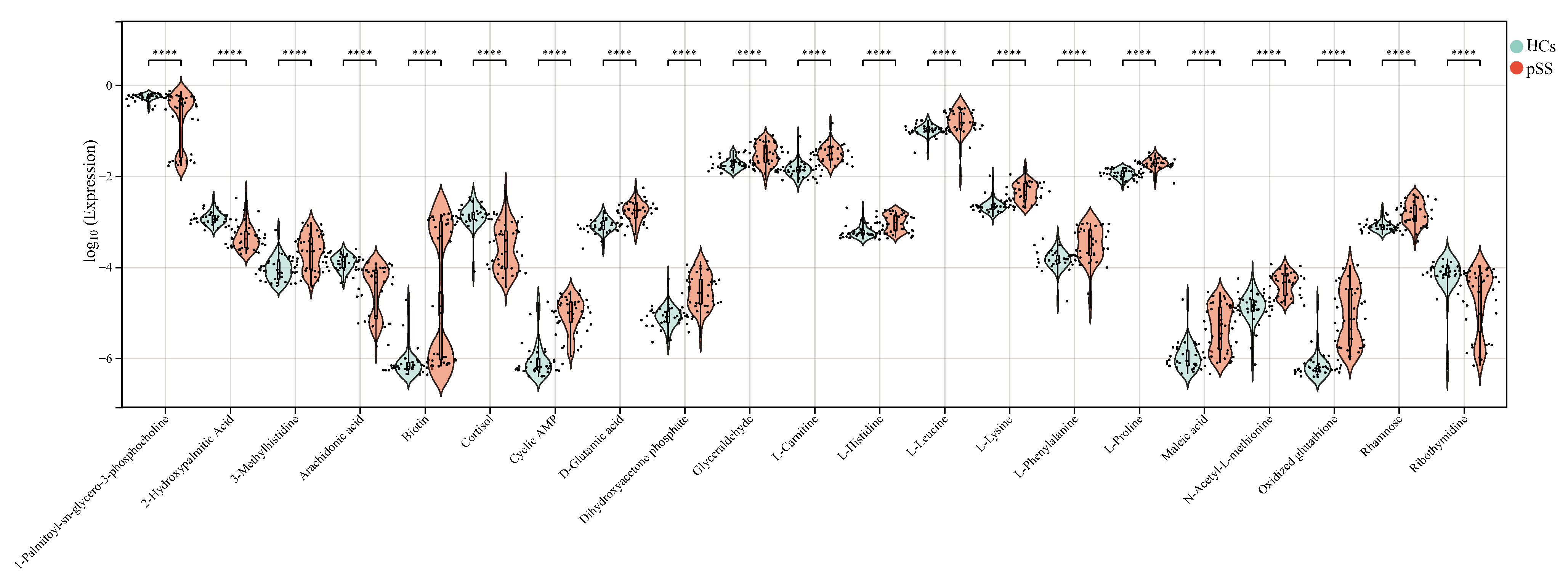


**Supplementary Figure S2**. The levels of 21 metabolites in patients with pSS compared with HCs by Mann-Whitney U test.


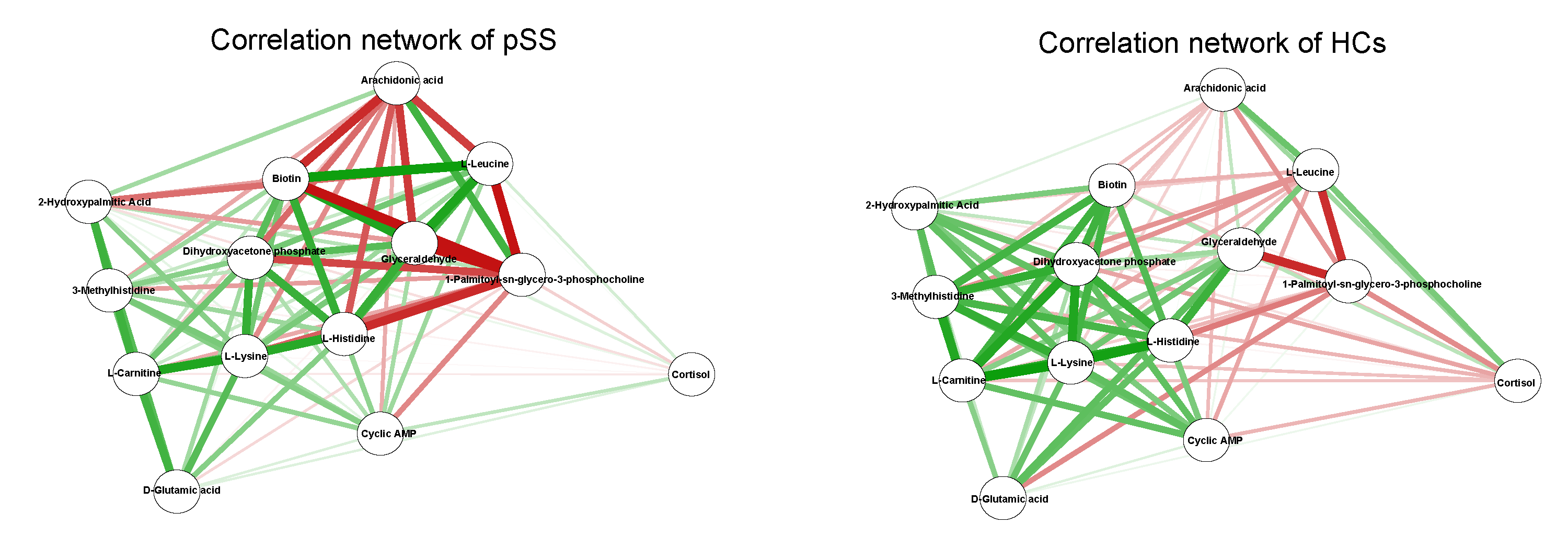


**Supplementary Figure S3**. Correlation network of 21 significantly different metabolites in HCs group and pSS group.


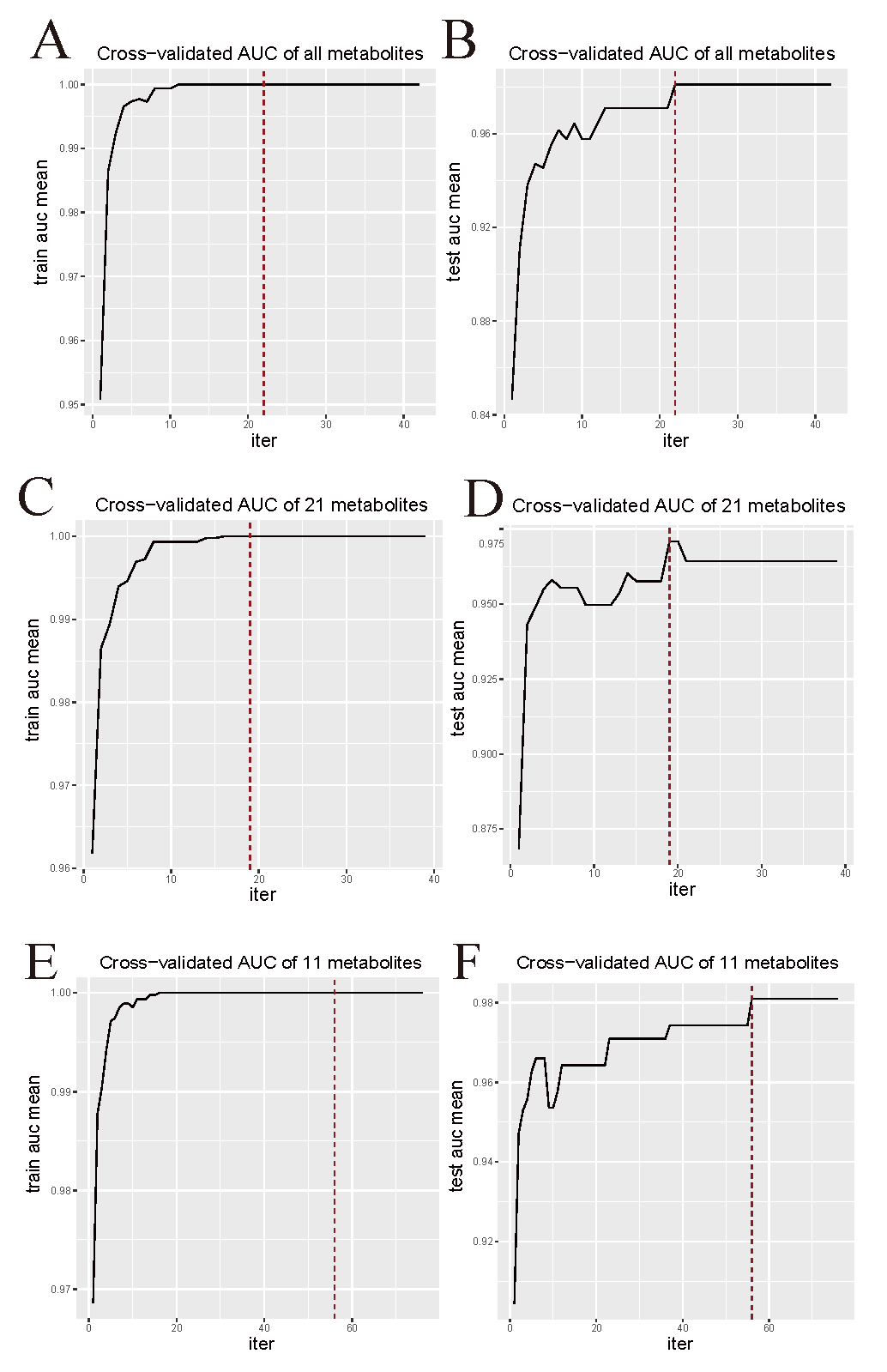


**Supplementary Figure S4**. AUC plots for the training (A) and testing (B) data of XGBoost on all metabolites (First round of machine learning), the best iteration is 22. AUC plots for the training (C) and testing (D) data of XGBoost on 21 metabolites with VIP value > 1.5 and FDR value < 0.05 (Second round of machine learning), the best iteration is 19. AUC plots for the training (E) and testing (F) data of XGBoost on important metabolites (Third round of machine learning), the best iteration is 56.


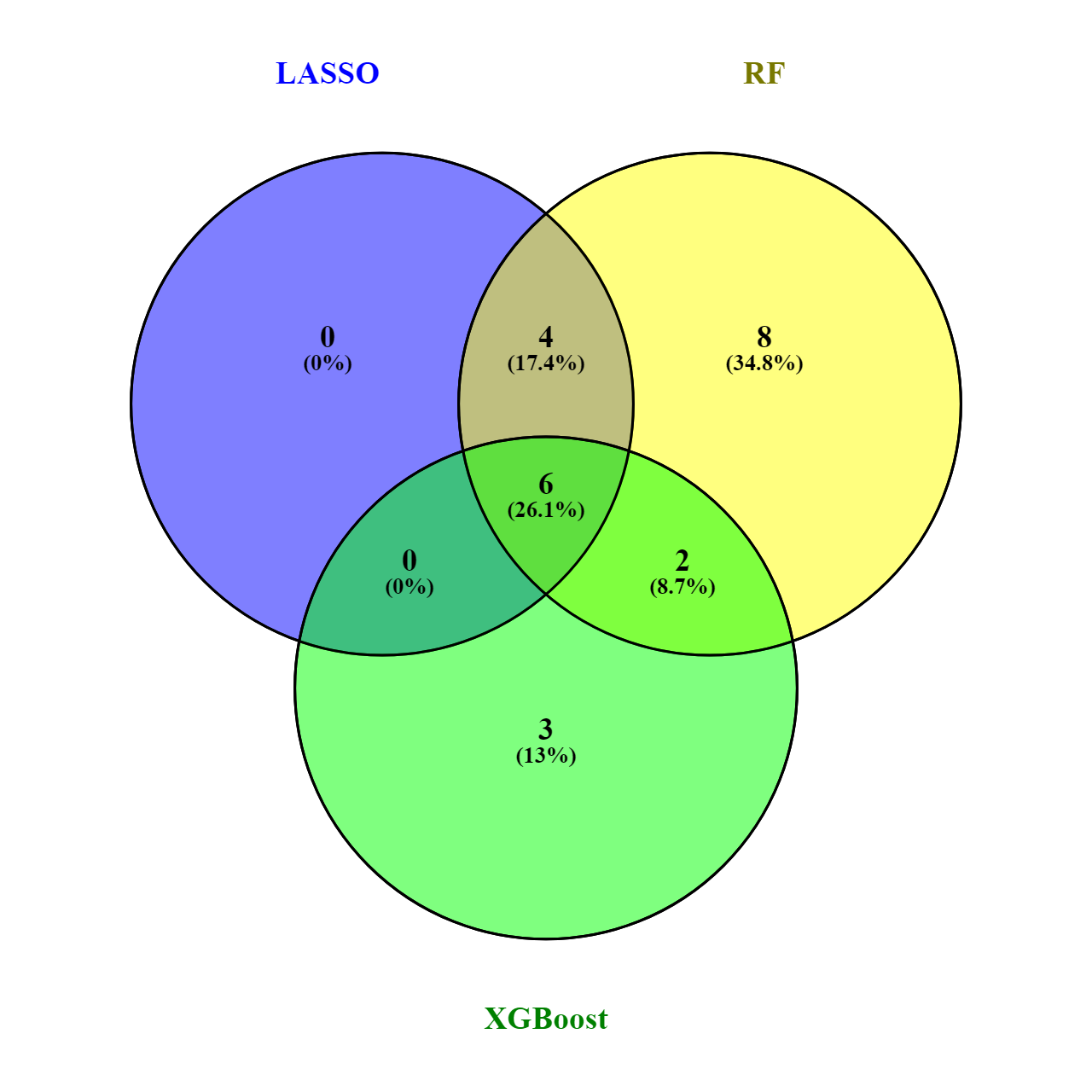


**Supplementary Figure** S5. The common important variables of three models, namely cyclic AMP (cAMP), cortisol, 2-Hydroxypalmitic acid, arachidonic acid, L-Carnitine and D-Glutamic acid.


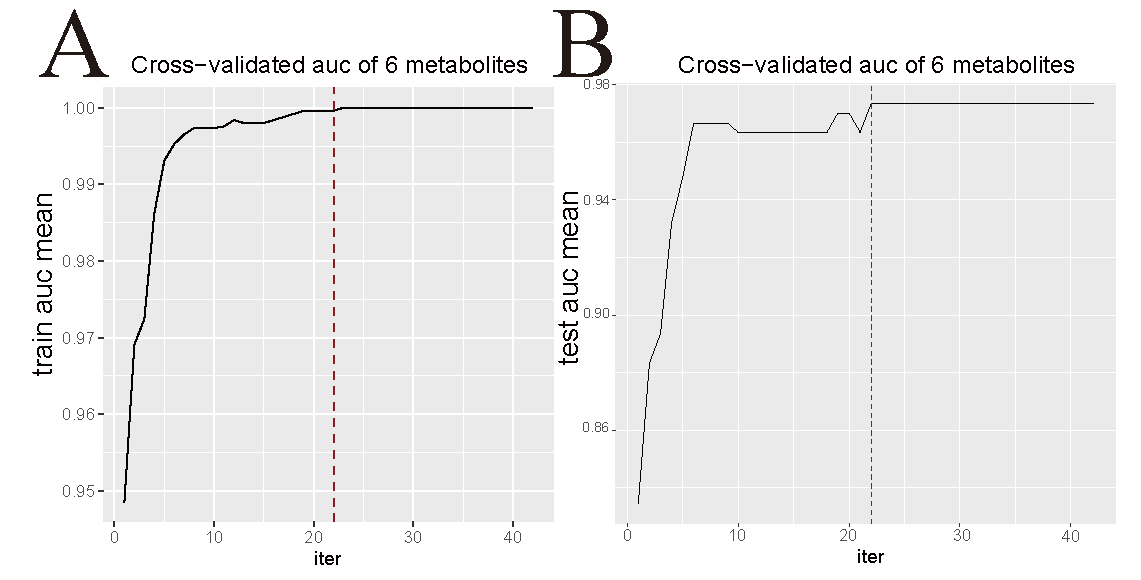


**Supplementary Figure** S6. AUC plots for the training (A) and testing (B) data of XGBoost on 6 metabolites (Fourth round of machine learning), the best iteration is 22.


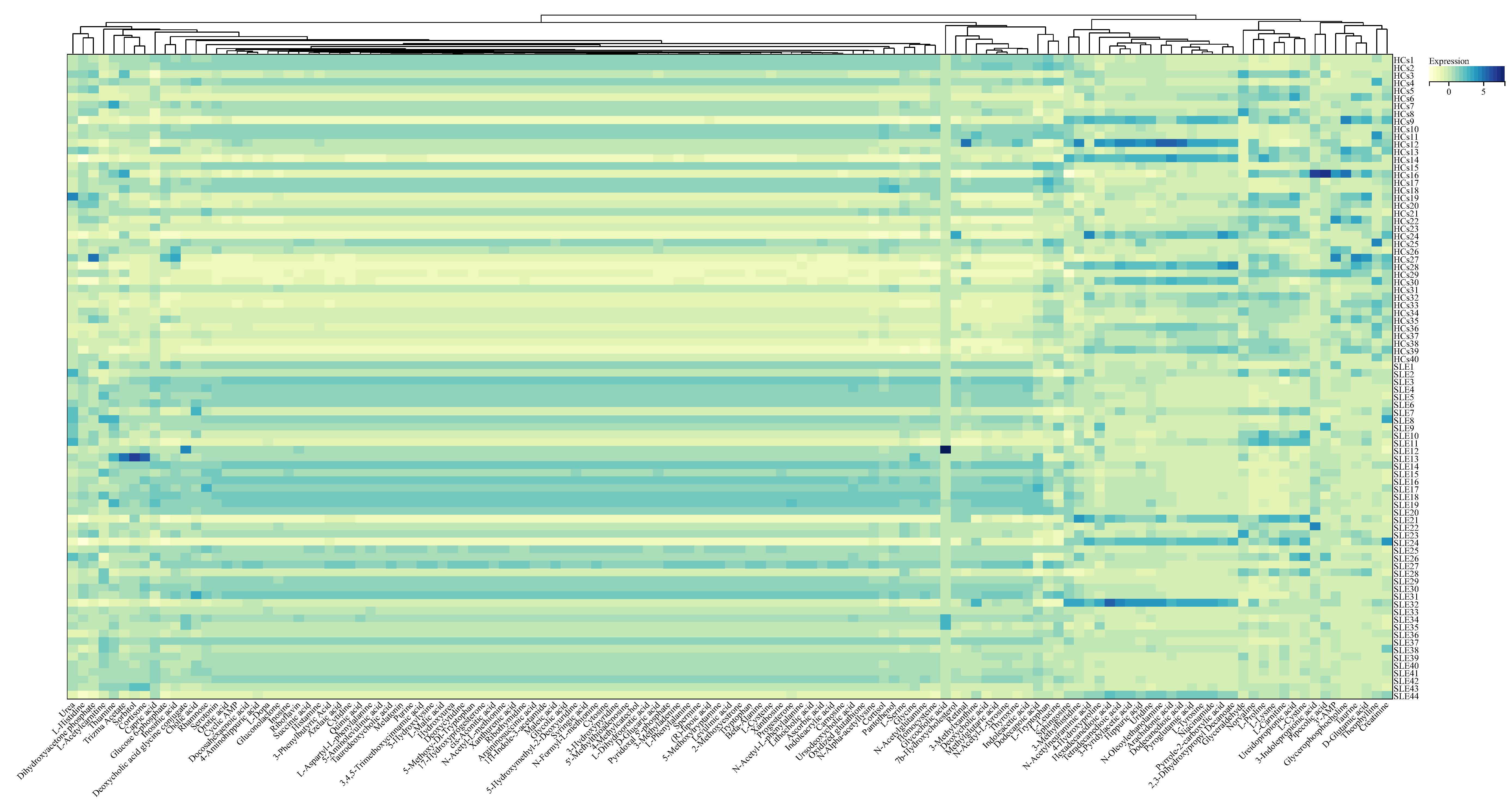


**Supplementary Figure S7**. Heat map of the SLE cohort, a total of 129 metabolites were identified in the metabolomic analysis of SLE cohort.

**Supplementary Table 1.** Demographics and clinical characteristics of pSS and HCs for the validation cohort.

| Variables | HCs (n=5) | pSS (n=6) | *P*value |
| --- | --- | --- | --- |
| Age (mean, range) | 44.60 ± 11.76 | 46.33 ± 13.84 | 0.696 |
| Gender (F/M) | 5/0 | 6/0 | >0.999 |
| Cholesterol (mmol/L) | 3.66 ± 0.40 | 5.16 ± 0.86 | **0.030** |
| ESR (mm/h) | 14.80 ± 3.11 | 28.83 ± 15.01 | **0.017** |
| CRP (mg/L) | 4.50 ± 1.09 | 10.05 ± 3.26 | **0.004** |
| Anti-SSA antibody (positive/negative) | 0/5 | 3/6 | 0.064 |
| Anti-SSB antibody (positive/negative) | 0/5 | 4/6 | 0.154 |
| ANA (positive/negative) | 0/5 | 4/6 | 0.154 |

Bold indicates statistical significance.

Abbreviations: pSS: primary Sjogren's syndrome; HCs, healthy contros; ESR: erythrocyte sedimentation rate; CRP: C-reactive protein; HCs: Healthy controls.

**Supplementary Table S2.** Demographics and clinical characteristics of SLE and HCs.

| Variables | SLE (n = 44) | HCs (n = 40) | *P*value |
| --- | --- | --- | --- |
| Age (years) | 33.82 ± 1.72 | 35.88 ± 1.33 | 0.174 |
| Gender (F/M) | 40 (90.91) | 35 (87.5) | 0.614 |
| Cholesterol (mmol/L) | 3.97 ± 0.2 | 3.48 ± 0.25 | 0.434 |
| ESR (mm/h) | 52.43 ± 5.13 | 17.25 ± 0.55 | **<0.001** |
| CRP (mg/L) | 20.73 ± 3.9 | 4.75 ± 0.27 | **0.004** |
| Anti-SSA antibody (positive/negative) | 18/26 | 3/37 | **<0.001** |
| Anti-SSB antibody (positive/negative) | 10/34 | 1/39 | **0.006** |
| ANA (positive/negative) | 31/13 | 2/38 | **<0.001** |

Bold indicates statistical significance.

Abbreviations: SLE, systemic lupus erythematosus; HCs, healthy contros; SLEDAI, systemic lupus erythematosus disease activity index; BMI, body mass index; WBC, white blood cell; PLT, platelet; ESR, erythrocyte sedimentation rate; CRP, C-reactive protein; ANA, antinuclear antibody; Ig, immunoglobulin.

**Supplementary Table S3.** Performance summary of different machine learning models based on all metabolites (First round of machine learning).

| Models | AUC | 95% CI | Sensitivity | Specificity | *P*-value |
| --- | --- | --- | --- | --- | --- |
| LASSO on train set | 1.000 | 1.000 - 1.000 | 100% | 100% | **<0.001** |
| LASSO on test set | 1.000 | 1.000 - 1.000 | 100% | 100% | **<0.001** |
| RF on train set | 0.982 | 0.902 - 1.000 | 100% | 96.3% | **<0.001** |
| RF on test set | 1.000 | 1.000 - 1.000 | 100% | 100% | **<0.001** |
| XGBoost on train set | 1.000 | 1.000 - 1.000 | 100% | 100% | **<0.001** |
| XGBoost on test set | 1.000 | 1.000 - 1.000 | 100% | 100% | **<0.001** |

Bold indicates statistical significance.

**Supplementary Table S4.** Performance summary of different machine learning models based on 21 metabolites with VIP value > 1.5 and FDR value < 0.05 (Second round of machine learning).

| Models | AUC | 95% CI | Sensitivity | Specificity | *P*-value |
| --- | --- | --- | --- | --- | --- |
| LASSO on train set | 1.000 | 1.000 - 1.000 | 100% | 100% | **<0.001** |
| LASSO on test set | 1.000 | 1.000 - 1.000 | 100% | 100% | **<0.001** |
| RF on train set | 0.982 | 0.902 - 1.000 | 100% | 96.3% | **<0.001** |
| RF on test set | 1.000 | 1.000 - 1.000 | 100% | 100% | **<0.001** |
| XGBoost on train set | 1.000 | 1.000 - 1.000 | 100% | 100% | **<0.001** |
| XGBoost on test set | 1.000 | 1.000 - 1.000 | 100% | 100% | **<0.001** |

Bold indicates statistical significance.

**Supplementary Table S5.** Performance summary of different machine learning models in their respective important metabolites (Third round of machine learning).

| Models | AUC | 95% CI | Sensitivity | Specificity | *P*-value |
| --- | --- | --- | --- | --- | --- |
| LASSO on train set | 1.000 | 1.000 - 1.000 | 100% | 100% | **<0.001** |
| LASSO on test set | 1.000 | 1.000 - 1.000 | 100% | 100% | **<0.001** |
| RF on train set | 0.982 | 0.902 - 1.000 | 100% | 96.3% | **<0.001** |
| RF on test set | 1.000 | 1.000 - 1.000 | 100% | 100% | **<0.001** |
| XGBoost on train set | 1.000 | 1.000 - 1.000 | 100% | 100% | **<0.001** |
| XGBoost on test set | 1.000 | 1.000 - 1.000 | 100% | 100% | **<0.001** |

Bold indicates statistical significance.

**Supplementary Table S6.** Performance summary of different machine learning models on 6 metabolites (Fourth round of machine learning).

| Models | AUC | 95% CI | Sensitivity | Specificity | *P*-value |
| --- | --- | --- | --- | --- | --- |
| LASSO on train set | 1.000 | 1.000 - 1.000 | 100% | 100% | **<0.001** |
| LASSO on test set | 0.955 | 0.772 - 0.999 | 100% | 90.9% | **<0.001** |
| LASSO on validation set | 0.900 | 0.576 - 0.997 | 100% | 80.0% | **<0.001** |
| RF on train set | 0.982 | 0.902 - 1.000 | 100% | 96.3% | **<0.001** |
| RF on test set | 1.000 | 1.000 - 1.000 | 100% | 100% | **<0.001** |
| RF on validation set | 1.000 | 1.000 - 1.000 | 100% | 100% | **<0.001** |
| XGBoost on train set | 1.000 | 1.000 - 1.000 | 100% | 100% | **<0.001** |
| XGBoost on test set | 1.000 | 1.000 - 1.000 | 100% | 100% | **<0.001** |
| XGBoost on validation set | 1.000 | 0.715 - 1.000 | 100% | 100% | **<0.001** |

Bold indicates statistical significance.

**Supplementary Table S7.** Performance summary of different machine learning models on 4 metabolites (metabolites with FDR and VIP values < 0.05 and > 2.0).

| Models | AUC | 95% CI | Sensitivity | Specificity | *P*-value |
| --- | --- | --- | --- | --- | --- |
| LASSO on train set | 0.945 | 0.848 - 0.988 | 96.43% | 92.59% | **<0.001** |
| LASSO on test set | 1.000 | 0.772 - 0.999 | 100% | 100% | **<0.001** |
| RF on train set | 0.964 | 0.875 - 0.996 | 96.43% | 96.3% | **<0.001** |
| RF on test set | 1.000 | 1.000 - 1.000 | 100% | 100% | **<0.001** |
| XGBoost on train set | 0.927 | 0.857 - 0.997 | 92.59% | 92.86% | **<0.001** |
| XGBoost on test set | 0.829 | 0.668 - 0.991 | 76.92% | 88.89% | **<0.001** |

Bold indicates statistical significance.

**Supplementary Table S8.** Performance summary of different machine learning models on 6 metabolites (Top 6 metabolites with VIP values).

| Models | AUC | 95% CI | Sensitivity | Specificity | *P*-value |
| --- | --- | --- | --- | --- | --- |
| LASSO on train set | 0.964 | 0.913 - 1.000 | 96.43% | 96.3% | **<0.001** |
| LASSO on test set | 1.000 | 0.772 - 0.999 | 100% | 100% | **<0.001** |
| RF on train set | 0.964 | 0.913 - 1.000 | 96.43% | 96.3% | **<0.001** |
| RF on test set | 1.000 | 1.000 - 1.000 | 100% | 100% | **<0.001** |
| XGBoost on train set | 0.983 | 0.949 - 1.000 | 100% | 96.55% | **<0.001** |
| XGBoost on test set | 1.000 | 1.000 - 1.000 | 100% | 100% | **<0.001** |

Bold indicates statistical significance. Top 6 metabolites with VIP values: arachidonic acid, cyclic AMP, L-Proline, N-Acetyl-L-methionine, cortisol and D-Glutamic acid.

**Supplementary Table S9.** Performance summary of different machine learning models on 6 metabolites (Top 6 metabolites with AUC values).

| Models | AUC | 95% CI | Sensitivity | Specificity | *P*-value |
| --- | --- | --- | --- | --- | --- |
| LASSO on train set | 0.964 | 0.913 - 1.000 | 96.43% | 96.3% | **<0.001** |
| LASSO on test set | 1.000 | 0.772 - 0.999 | 100% | 100% | **<0.001** |
| RF on train set | 0.982 | 0.945 - 1.000 | 100% | 96.3% | **<0.001** |
| RF on test set | 1.000 | 1.000 - 1.000 | 100% | 100% | **<0.001** |
| XGBoost on train set | 0.983 | 0.949 - 1.000 | 100% | 96.55% | **<0.001** |
| XGBoost on test set | 1.000 | 1.000 - 1.000 | 100% | 100% | **<0.001** |

Bold indicates statistical significance. Top 6 metabolites with AUC values: oxidized glutathione, cyclic AMP, L-Carnitine, L-Proline, N-Acetyl-L-methionine and Cortisol.
